# Chromosome 3p Inverted Duplication with Terminal Deletion: Second Postnatal Case Report with Additional Clinical Features

**DOI:** 10.1155/2019/5384295

**Published:** 2019-07-25

**Authors:** Jacquelyn D. Riley, Catherine M. Stefaniuk, Francine Erenberg, Angelika L. Erwin, Lauren Palange, Caroline Astbury

**Affiliations:** ^1^Molecular Pathology Section, Pathology and Laboratory Medicine Institute, USA; ^2^Department of Pathology and Laboratory Medicine, University of Cincinnati Health, Cincinnati, Ohio, USA; ^3^Pediatric Cardiology, Pediatric Institute, USA; ^4^Center for Personalized Genetic Healthcare, Genomic Medicine Institute, Cleveland Clinic, Cleveland, Ohio, USA

## Abstract

Distal deletions and duplications of 3p are individually well-characterized chromosome abnormalities. Here, we report an inverted duplication of 3p with an adjacent terminal 3p deletion in a 17-month-old girl who had prenatal intrauterine growth restriction and cardiac defects. Other findings included hemangiomas, neutropenia, umbilical hernia, hypotonia, gross motor delay, microcephaly, and ptosis. Family history was noncontributory. Microarray analysis revealed a 5.37 Mb deletion of chromosome bands 3p26.1 to 3p26.3 and a 13.68 Mb duplication of 3p24.3 to 3p26.1. FISH analysis confirmed that the duplication was inverted. Upon literature review, only one postnatal patient and one second trimester pregnancy have been reported with this finding. Many of our patient's features are present in both 3p deletion and 3p duplication syndromes, including congenital heart disease, growth restriction, microcephaly, hypotonia, and developmental delay. Our patient has additional features not commonly reported in 3p deletion or duplication patients, such as aortic dilation, hemangiomas, and neutropenia. The identification of this patient contributes to additional understanding of features associated with concurrent deletion and inverted duplication in the distal 3p chromosome. This report may assist clinicians working with patients who have constellations of similar features or similar cytogenomic abnormalities.

## 1. Introduction

The medical literature contains numerous reports of patients with either duplications or deletions within chromosome 3p21-p26. Early reports were based on methods with limited resolution; chromosomal breakpoints were less precise and small imbalances may have gone undetected. Later cases based on fluorescence* in situ* hybridization (FISH) probes and array comparative genomic hybridization (aCGH) refined regions of imbalance. However, since many cases were caused by inherited translocations or other rearrangements that also resulted in partial monosomy of a second chromosome, it was difficult to distinguish the clinical findings due to loss or gain of 3p21-p26 from those due to a chromosome imbalance elsewhere in the genome.

A report in 1978 first summarized the clinical features seen in eight cases of partial trisomy 3p, highlighting distinctive features of microcephaly, frontal bossing, square-shaped face, hypertelorism/telecanthus, prominent cheeks, temporal indentation, large mouth with micrognathia/retrognathia, penile hypoplasia, congenital heart defects, and mental delay [1]. Conte et al. [2] updated the literature review and listed additional major features including brachycephaly, short nose, prominent philtrum, low-set and/or dysmorphic ears, downturned mouth corners, short neck, abnormal muscle tone, genitourinary malformations, and hypoplastic genitalia in females. Additional case reports described patients with smaller duplications that notably lacked dysmorphism or major congenital anomalies [3–5].

The first report of a pure distal 3p deletion was in the 1970s in the Netherlands of a male child [6]. With additional reports, a syndromic constellation of features emerged, which included growth restriction, microcephaly with trigonocephaly, narrow forehead with prominent metopic suture, ptosis, inner epicanthic folds, upslanting palpebral fissures, synophrys, small nose with anteverted nostrils, low set/poorly shaped ears (possibly with preauricular pits/fistulas), postaxial polydactyly, congenital heart defects, renal malformations, gastric malformations, hypotonia, and mental and psychomotor delays [7]. Again, recent reports demonstrated a wider spectrum of phenotype, particularly among patients with smaller deletions of 3p [8–10]. A summary of literature and clinical synopsis of Chromosome 3pter-3p25 Deletion Syndrome is available in Online Mendelian Inheritance in Man (OMIM #613792) [11].

Here, we present a patient with both a duplication and a deletion of chromosome 3p, who is only the third patient described to have such findings result from an inverted duplication with a terminal deletion. Our patient has clinical findings consistent with both partial monosomy and partial trisomy of 3p, as well as features that are not commonly reported in either condition.

## 2. Case Presentation

Our patient, a Caucasian female last evaluated at 17 months of age, came to medical attention prenatally due to placental calcifications, intrauterine growth restriction, and concern for cardiac defect. She was born at 35-weeks and 5-days gestation via induced vaginal delivery secondary to decreased fetal movement and nonreactive nonstress test. At the time of her birth, her mother was 29 years old and her father was 37 years old. Apgar scores were 6, 9, and 10, at 1, 5, and 10 minutes, respectively. She had respiratory distress and feeding difficulties, requiring CPAP in the delivery room and an NG tube during her hospitalization. She was diagnosed with a mildly dilated aortic root and ascending aorta, dilated pulmonary artery, atrial and ventricular septal defects, mitral valve prolapse with mitral regurgitation, reducible umbilical hernia, and an urachal remnant. Placental pathology was consistent with placenta accreta. Upon discharge at 12 days of age, weight was 1825 grams (birth weight: 1690 grams, less than 3^rd^ centile), length was 41.6 cm (3^rd^-10^th^ centile), and head circumference was 31cm (3^rd^-10^th^ centile). Subsequent phenotypic findings included hemangiomas of right flank and liver, neutropenia (later determined to be autoimmune in nature), transient anemia (resolved within a few months after intravenous iron administration and a blood transfusion), umbilical hernia, hypotonia, global developmental delay, and myopic astigmatism. Additional physical features were a supernumerary nipple, ptosis, prominent epicanthal folds, broad nasal bridge, and smooth philtrum. At 17 months, her weight was in the second centile (7.9 kg), height was in the eighth centile (75.5 cm), and head circumference was in the first centile (43 cm). Her parents reported significant sleep issues since early infancy, manifesting in difficulty going to sleep as well as frequent awakening. She received behavioral therapy, which led to slight improvement of these symptoms. In addition, our patient was diagnosed with hypothyroidism which is controlled on levothyroxine. Family history was noncontributory and there was no known consanguinity. To evaluate our patient's findings, microarray analysis was pursued.

**Figure 1:**
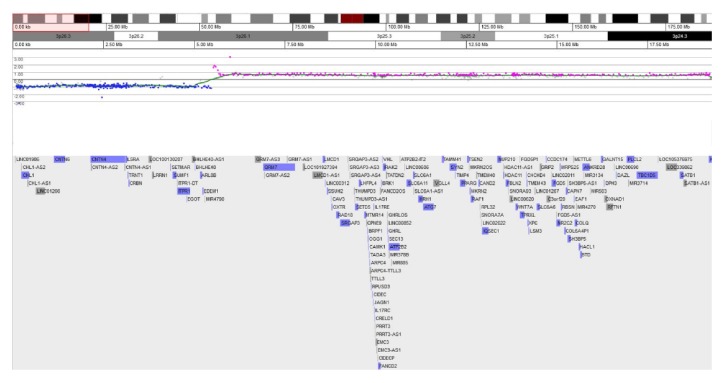
Copy number changes in chromosome 3p. Copy loss in bands 3p26.3-p26.1 (genomic coordinates: chr3:101072_5470617), estimated size 5.37-5.53 Mb. Copy gain in bands 3p26.1-p24.3 (genomic coordinates: chr3:5530662_19214536), estimated size 13.68-13.75 Mb. GRCh37/hg19 (Feb. 2009).

aCGH was performed on DNA isolated from peripheral blood lymphocytes using the GGXChip + SNP v1.0 array platform (Agilent Technologies, Santa Clara, CA, USA). The CGH array included 107,000 probes with an average resolution backbone of 80 kilobases and of 20 kilobases in targeted regions. A copy number variation is defined as being present if 5 or more consecutive probes either above a derivative log ratio (DLR) of 0.5 (copy number gain) or below a DLR of -0.5 (copy number loss) are observed. The SNP platform included 60,000 probes with an average spacing of 50 kb. Regions of homozygosity of greater than 10 Mb (or 8 Mb if telomeric) are reported. The reference genome utilized was GRCh37/hg19 (Feb. 2009). Data was analyzed through CytoGenomics (Agilent Technologies) and visualized through Genoglyphix® (PerkinElmer, Waltham, MA, USA). aCGH analysis revealed a 5.37 Mb deletion encompassing 3p26.1-3p26.3, containing a total of 22 genes (13 OMIM genes and 9 other genes) (Figure 1). In addition, there was a 13.68 Mb duplication encompassing 3p24.3-3p26.1, which contained a total of 126 genes (70 OMIM genes and 56 other genes) (Figure 1). There was an approximately 10 kb gap between the last probe in the inverted duplication region and the first disomic probe in 3p24.3. There was an approximately 60 kb gap between the last probe in the deleted region in 3p26.1 and the first probe in the duplicated region (also in 3p26.1), with no disomic probes between them. The possibility of a normal, disomic region within this 60 kb gap could not be ruled out and additional testing to elucidate this was not pursued since there was no clear clinical benefit.

**Figure 2:**
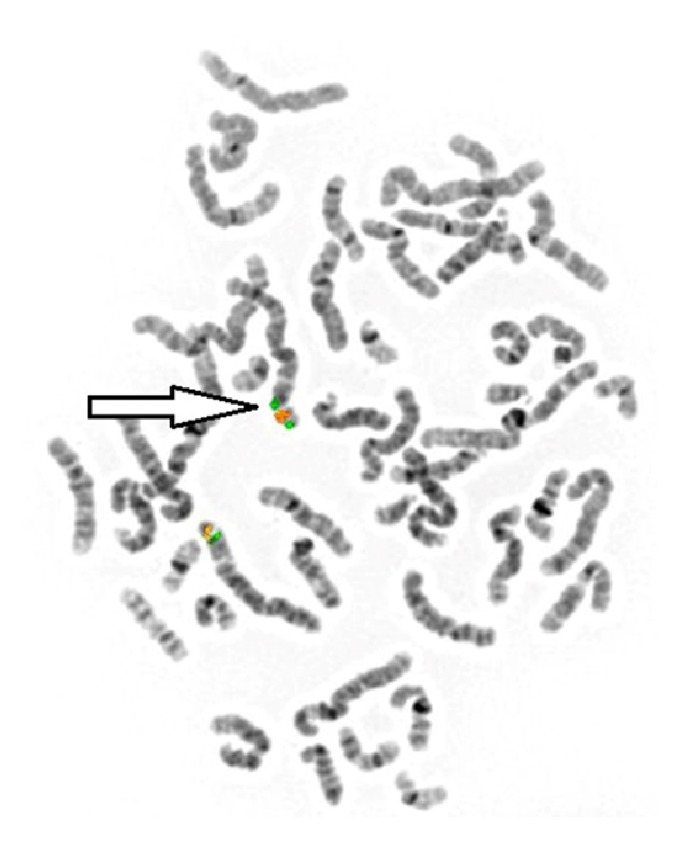
FISH analysis demonstrating the 3p26.1 to 3p24.3 inverted duplication. The probes, RP11-451A20 at 3p24.3 (green) and RP11-266J6 at 3p25.3 (red), demonstrated 2 signals for each in a green, red, red, green pattern.

To further clarify the microarray findings, FISH studies were performed on metaphase cells. Probes specific to the chromosomal regions 3p26.3 (RP11-204C23; green), 3p25.3 (RP11-266J6; red), and 3p24.3 (RP11-451A20; green) (Empire Genomics LLC, Williamsville, NY, USA) were used to characterize the structural rearrangement. For analysis of parental chromosomes, the same 3p26.3 and 3p25.3 probes were used to determine whether the structural rearrangement was inherited. FISH analysis of the proband demonstrated a single copy of the RP11-204C23 probe at 3p26.3 and three copies of the RP11-266J6 probe at 3p25.3. Further analysis with the RP11-451A20 probe at 3p24.3 and RP11-266J6 at 3p25.3 demonstrated 2 signals for each of the probes in a green, red, red, green pattern on the derivative chromosome 3 (Figure 2). The orientation of the probe signals on the abnormal chromosome 3 confirmed that the duplication was inverted. Parental FISH studies demonstrated two copies of RP11-204C23 at 3p26.3 and RP11-266J6 at 3p25.3 in the correct orientation and location, thus confirming* de novo* occurrence. The proband's cytogenetic findings were reported as arr[GRCh37] 3p26.3p26.1(101072_5470617)x1 dn,3p26.1p24.3(5530662_19214536)x3 dn.

## 3. Discussion

Upon extensive literature review, this is only the third reported case of an inverted duplication of chromosome 3p with an adjacent terminal deletion of 3p. The first patient described had a larger duplication and the limited clinical description included features consistent with partial trisomy 3p syndrome including characteristic facial dysmorphism, cleft lip and palate, and short neck, along with features that are common in both deletions and duplications of 3p such as developmental delay, microcephaly, and congenital heart disease [12]. She was noted to lack features specific to the 3p deletion. The authors used a combination of traditional and molecular cytogenetics techniques and her karyotype was reported as 46,XX,invdup(3)(qter > p26::p26- > p21.3::p26). This also allowed them to explore the mechanisms by which the partial trisomy and partial monosomy originated, with results suggestive of a symmetric or asymmetric recombination process with U-type exchange resulting in a dicentric chromosome.

A prenatal patient has also been described, with a severe phenotype detected on ultrasound at 20 weeks' gestation [13]. The fetus had holoprosencephaly, midline cleft lip and palate, and congenital heart defect along with a lumbosacral meningomyelocele. The pregnancy was terminated at 22 weeks' gestation. Chromosome and FISH analysis revealed a karyotype of 46,XY,der(3)del(3)(p26)dup(3)(p26p21.3). Although rare, 3p duplication cases have previously been associated with severe holoprosencephaly [14–16].

Our patient has several features that are reported in both 3p deletion and 3p duplication syndromes, including congenital heart disease, growth restriction, microcephaly, hypotonia, and developmental delay (Table 1). Interestingly, our patient has minimal facial dysmorphism. She is noted to have ptosis, epicanthal folds, and a broad nasal bridge (all associated with 3p deletion) but lacks many other facial characteristics typically noted in either syndrome. Unlike the previously reported patients, her phenotype more closely resembles the 3p deletion syndrome than the 3p duplication syndrome. Further, our patient has additional features not commonly reported in these syndromes, such as aortic and arterial dilation, neutropenia, and cutaneous and hepatic hemangiomas.

Congenital heart disease is commonly reported in both 3p deletions and 3p duplications and most cases involve septal and/or valvular abnormalities. No other reports of aortic dilation or pulmonary artery dilation were found among 3p deletion or duplication patients except when the* TGFBR2* gene (3p24.1) was involved. This gene is not involved in the chromosomal rearrangement in our patient.

Our patient also has anemia and neutropenia. There are a number of genes in the 3p region associated with hematologic disorders. Pathogenic variants in* TRNT2* (3p26.2) can lead to autosomal recessive sideroblastic anemia with B-cell immunodeficiency or erythrocytic microcytosis. This gene is in our patient's deleted region so an autosomal recessive condition is plausible if the remaining copy of the gene has a pathogenic variant. The* FANCD2* gene (3p25.3) is in the duplicated region for our patient. Fanconi anemia has autosomal recessive inheritance so a duplication of this gene would not likely result in a similar phenotype. Pathogenic variants in* JAGN1*, also in the 3p25.3 duplicated region, lead to severe congenital neutropenia, but this condition also has autosomal recessive inheritance.

Although not commonly described in 3p duplication syndrome, hemangiomas have been reported in patients with abnormalities involving this chromosomal region. In one report, a patient was suspected to have Opitz C (trigonocephaly) syndrome and her clinical features included a forehead hemangioma/nevus flammeus. Subtelomeric FISH analysis demonstrated duplication of 3p26.3, secondary to a translocation [17]. This is difficult to correlate with our patient, who has a deletion, not a duplication, at 3p26.3. There was also a report of a female with dorsal midline cutaneous hemangiomas with a dup(3)(pter*⟶*p26::p22*⟶*p26::p26*⟶*qter) [3], which overlaps with and is larger than our patient's duplication. The duplicated region in our patient also includes* VHL*, a tumor suppressor gene. Haploinsufficiency of this gene is associated with von Hippel-Lindau syndrome, a cancer predisposition syndrome in which liver hemangiomas have been reported. However, to our knowledge there are no reports of duplications of* VHL* resulting in similar phenotypes.

Though this is only the second postnatal case of an inverted duplication with terminal deletion of 3p, such rearrangements have been reported in almost all chromosomes. With the increased resolution of FISH, microarrays, and next-generation sequencing, inverted duplications with terminal deletions are being more frequently identified [18]. The mechanism of the relatively common recurrent rearrangement that results in inverted duplication and terminal deletion of chromosome 8p has been well-studied [19, 20]. However, the mechanisms causing nonrecurrent rearrangements have been more challenging to characterize. To the best of our knowledge, there are no segmental duplications that align with the breakpoints of the rearrangement identified in our patient, with the nearest being approximately 250-260 kb away from each presumed breakpoint. Previous work proposed that, following a double-stranded DNA break resulting in loss of a terminal segment, the repair process involved a U-type exchange [21]. More recent work suggests a repair process that includes a fold-back mechanism by which the free chromosome end pairs with itself and DNA synthesis fills in the gap [22]. After replication, an unstable dicentric chromosome is created that undergoes a second double-stranded break, resulting in one chromosome with a duplication and a terminal deletion and a second chromosome with a terminal deletion alone.

Structural abnormalities of chromosome 3p have been described and genotype-phenotype correlations have led to the identification of several critical regions and candidate genes [4, 11, 23]. However, much remains unknown, including the full phenotypic spectrum associated with deletions and duplications in these regions and the factors associated with variability among patients with similar copy number changes. In the Jenderny [12] and Kennedy [13] cases, the patients had disparate phenotypes but neither displayed clinical features unique to 3p deletion syndrome. Our patient has features seen in both 3p deletion and 3p duplication syndromes, as well as features not commonly described in either syndrome thus far. The identification of this patient contributes to further understanding of features associated with concurrent duplication and deletion in the distal short arm of chromosome 3.

## Figures and Tables

**Table 1:** Comparison of phenotypic features of patients with chromosome 3p duplication with terminal deletion.

Phenotypic Features	Common Features of 3pterp25 Deletion Syndrome^†^	Common Features of Chromosome 3pterp24 Duplications^*∗*^	Our Patient	Jenderny Patient	Kennedy Patient
			46XX,der(3)del(3)(p26.3p 26.1)dup(3)(p24.3p26.1)	46,XX,invdup(3)(qter > p26::p26- > p21.3::p26)	46,XY,der(3)del(3)(p26)d up(3)(p26p21.3)
Congenital heart defects		+	+	+	+
Dilated aorta, pulmonary artery			+		
Umbilical hernia			+		
Cleft lip and palate			-	+	+
Holoprosencephaly			-		+
Meningomyelocele			-		+
Microcephaly	+		+	+	
Supernumerary nipple			+		
Hemangiomas			+		
Trigonocephaly	+		-		
Micro/retrognathia	+		-	+	
Growth retardation	+		+	-	
Feeding difficulties			+		
Neutropenia, autoimmune			+(resolved)		
Anemia			+(resolved)		
Hypothyroidism			+		
Hypotonia	+		+		
Developmental delay/psychomotor retardation	+	+	+	+	
Intellectual disability	+	+		+	
Sleep abnormalities			+		
Astigmatism			+		
Myopia			+		
Ptosis	+		+(resolved)	-	
Prominent epicanthal folds	1		1		
Square-shaped face		+	-	+	
Frontal bossing		+	-	+	
Temporal indentation			-	+	
Prominent cheeks			-	+	
Hypertelorism	+	+	+	+	
Downslanting palpebral fissures	+		-		
Abnormal nose	+		+		
Abnormal philtrum	+		+		
Down-turned corners of the mouth		+	-	+	
Prominent middle upper lip		+	-		
Eversion of lips			-	+	
Low-set/dysmorphic ears	+	+	+		
Short neck		+	-	+	

^†^Based on features reported in ≥50% of patients reviewed by Fernandez et al. 2008 [9].

^*∗*^Based on features reported in ≥50% of patients (Case Numbers 12-14, 20, 31, 35, 43, 44) reviewed by Conte et al. 1995 [2].

Blanks indicate features not reported as positive or negative findings in individual patients.

## References

[1] Yunis J. J. (1978). Trisomy for the Distal End of the Short Arm of Chromosome 3. *American Journal of Diseases of Children*.

[2] Conte R. A., Pitter J. H., Verma R. S. (1995). Molecular characterization of trisomic segment 3p24.1→3pter: a case with review of the literature. *Clinical Genetics*.

[3] Smeets E., Vandenbossche L., Fryns J. P. (2001). Partial distal trisomy 3p. A partial autosomal trisomy without major dysmorphic features. *Journal of Genetic Counseling*.

[4] Bittel D. C., Kibiryeva N., Dasouki M., Knoll J. H. M., Butler M. G. (2006). A 9-year-old male with a duplication of chromosome 3p25.3p26.2: clinical report and gene expression analysis. *American Journal of Medical Genetics Part A*.

[5] Natera-de Benito D., García-Pérez M. A., Martínez-Granero M. Á., Izquierdo-López L. (2014). A patient with a duplication of chromosome 3p (p24.1p26.2): a comparison with other partial 3p trisomies. *American Journal of Medical Genetics Part A*.

[6] Verjaal M. (1978). A patient with a partial deletion of the short arm of chromosome 3. *American Journal of Diseases of Children*.

[7] Schwyzer U., Binkert F., Caflisch U., Baumgartner B., Schinzel A. (1987). Terminal deletion of the short arm of chromosome 3, del(3pter-p25): a recognizable syndrome. *Helvetica Paediatrica Acta*.

[8] Knight L. A., Yong M. H., Tan M., Ng I. S. (1995). Del(3) (p25.3) without phenotypic effect.. *Journal of Medical Genetics*.

[9] Fernandez T. V., García-González I., Mason C. E. (2008). Molecular characterization of a patient with 3p deletion syndrome and a review of the literature. *American Journal of Medical Genetics Part A*.

[10] Tassano E., Biancheri R., Denegri L. (2014). Heterozygous deletion of CHL1 gene: detailed array-CGH and clinical characterization of a new case and review of the literature. *European Journal of Medical Genetics*.

[11] Chromosome 3pter-p25 Deletion Syndrome (#613792). 2017. Online Mendelian Inheritance in Man (OMIM). Accessed September 25, 2018, http://omim.org/

[12] Jenderny J., Poetsch M., Hoeltzenbein M., Friedrich U., Jauch A. (1998). Detection of a concomitant distal deletion in an inverted duplication of chromosome 3. Is there an overall mechanism for the origin of such duplications/deficiencies?. *European Journal of Human Genetics*.

[13] Kennedy D., Silver M. M., Winsor E. J. T. (2000). Inverted duplication of the distal short arm of chromosome 3 associated with lobar holoprosencephaly and lumbosacral meningomyelocele. *American Journal of Medical Genetics*.

[14] Martin N. J., Steinberg B. G., Opitz J. M. (1983). The dup(3)(p25 → pter) syndrome: a case with holoprosencephaly. *American Journal of Medical Genetics*.

[15] Gillerot Y., Hustin J., Koulischer L., Viteux V., Neri G., Opitz J. M. (1987). Prenatal diagnosis of a dup(3p) with holoprosencephaly. *American Journal of Medical Genetics*.

[16] Kurtzman D. N., Van Dyke D. L., Rich C. A., Weiss L., Opitz J. M., Reynolds J. F. (1987). Duplication 3p21→3pter and cyclopia. *American Journal of Medical Genetics*.

[17] McGaughran J., Aftimos S., Oei pp. (2000). Trisomy of 3pter in a patient with apparent c (trigonocephaly) syndrome. *American Journal of Medical Genetics*.

[18] Zuffardi O., Bonaglia M., Ciccone R., Giorda R. (2009). Inverted duplications deletions: underdiagnosed rearrangements??. *Clinical Genetics*.

[19] Giglio S., Broman K. W., Matsumoto N. (2001). Olfactory receptor–gene clusters, genomic-inversion polymorphisms, and common chromosome rearrangements. *American Journal of Human Genetics*.

[20] Buysse K., Antonacci F., Callewaert B. (2009). Unusual 8p inverted duplication deletion with telomere capture from 8q. *European Journal of Medical Genetics*.

[21] Rowe L. R., Lee J., Rector L. (2009). U-type exchange is the most frequent mechanism for inverted duplication with terminal deletion rearrangements. *Journal of Medical Genetics*.

[22] Hermetz K. E., Newman S., Conneely K. N. (2014). Large inverted duplications in the human genome form via a fold-back mechanism. *PLoS Genetics*.

[23] Chabchoub E., Mchils G., Vermeesch J. R., De Cock P., Lagae L., Fryns J. P. (2010). Duplication of the VHL and IRAK2 genes in a patient with mental retardation/multiple congenital anomalies, epilepsy and ectomorphic habitus. *Journal of Genetic Counseling*.

