# Corrigendum to “Chromosome 3p Inverted Duplication with Terminal Deletion: Second Postnatal Case Report with Additional Clinical Features”

**DOI:** 10.1155/2019/4361630

**Published:** 2019-11-24

**Authors:** Jacquelyn D. Riley, Catherine M. Stefaniuk, Francine Erenberg, Angelika L. Erwin, Lauren Palange, Caroline Astbury

**Affiliations:** ^1^Molecular Pathology Section, Pathology and Laboratory Medicine Institute, Cleveland Clinic, Cleveland, OH, USA; ^2^Department of Pathology and Laboratory Medicine, University of Cincinnati Health, Cincinnati, OH, USA; ^3^Pediatric Cardiology, Pediatric Institute, Cleveland Clinic, Cleveland, OH, USA; ^4^Center for Personalized Genetic Healthcare, Genomic Medicine Institute, Cleveland Clinic, Cleveland, OH, USA

In the article titled “Chromosome 3p Inverted Duplication with Terminal Deletion: Second Postnatal Case Report with Additional Clinical Features” [1], the first and third affiliations were incomplete. The corrected authors' list and affiliations are shown above.
